# Locus Coeruleus and Tuberomammillary Nuclei Ablations Attenuate Hypocretin/Orexin Antagonist-Mediated REM Sleep^1^,^2^,^3^

**DOI:** 10.1523/ENEURO.0018-16.2016

**Published:** 2016-03-21

**Authors:** Michael D. Schwartz, Alexander T. Nguyen, Deepti R. Warrier, Jeremiah B. Palmerston, Alexia M. Thomas, Stephen R. Morairty, Thomas C. Neylan, Thomas S. Kilduff

**Affiliations:** 1 Biosciences Division, Center for Neuroscience, SRI International, Menlo Park, California 94025; 2 UCSF San Francisco VA Medical Center/NCIRE, San Francisco, California 94121

**Keywords:** arousal, hypnotics, insomnia, monoamine, orexin, paradoxical sleep

## Abstract

Hypocretin 1 and 2 (Hcrts; also known as orexin A and B), excitatory neuropeptides synthesized in cells located in the tuberal hypothalamus, play a central role in the control of arousal. Hcrt inputs to the locus coeruleus norepinephrine (LC NE) system and the posterior hypothalamic histaminergic tuberomammillary nuclei (TMN HA) are important efferent pathways for Hcrt-induced wakefulness. The LC expresses Hcrt receptor 1 (HcrtR1), whereas HcrtR2 is found in the TMN. Although the dual Hcrt/orexin receptor antagonist almorexant (ALM) decreases wakefulness and increases NREM and REM sleep time, the neural circuitry that mediates these effects is currently unknown. To test the hypothesis that ALM induces sleep by selectively disfacilitating subcortical wake-promoting populations, we ablated LC NE neurons (LCx) or TMN HA neurons (TMNx) in rats using cell-type-specific saporin conjugates and evaluated sleep/wake following treatment with ALM and the GABA_A_ receptor modulator zolpidem (ZOL). Both LCx and TMNx attenuated the promotion of REM sleep by ALM without affecting ALM-mediated increases in NREM sleep. Thus, eliminating either HcrtR1 signaling in the LC or HcrtR2 signaling in the TMN yields similar effects on ALM-induced REM sleep without affecting NREM sleep time. In contrast, neither lesion altered ZOL efficacy on any measure of sleep–wake regulation. These results contrast with those of a previous study in which ablation of basal forebrain cholinergic neurons attenuated ALM-induced increases in NREM sleep time without affecting REM sleep, indicating that Hcrt neurotransmission influences distinct aspects of NREM and REM sleep at different locations in the sleep–wake regulatory network.

## Significance Statement

The hypocretin/orexin (Hcrt) system powerfully regulates arousal in part by excitatory projections to wake-promoting cell groups in the posterior hypothalamus and brainstem. Cell-type-specific ablations of the locus coeruleus norepinephrine (LC NE) neurons or the tuberomammillary histamine (TMN HA) neurons decreased the hypnotic efficacy of the dual Hcrt receptor antagonist Almorexant, while having no effect on sleep promotion by the GABA receptor modulator zolpidem. Lesioning the LC or TMN attenuated almorexant-induced REM sleep without affecting NREM sleep time. Lesions exerted similar effects independently of the Hcrt receptor type expressed in each region, suggesting that the site of action, not just the specific receptor or receptors targeted, is a key determinant of how Hcrt receptor antagonism facilitates sleep.

## Introduction

Hypocretin-1 and -2 (Hcrts; also known as orexin-A and -B), excitatory neuropeptides synthesized in neurons located in the tuberal hypothalamus, are involved in metabolism, feeding, reward, addiction, and sleep–wake control (Ohno and Sakurai, 2008). Hcrt neurons are wake-active (Estabrooke et al., 2001; Lee et al., 2005). Hcrt administration (Bourgin et al., 2000; Morairty et al., 2011) or optogenetic stimulation of Hcrt neurons (Adamantidis et al., 2007; Carter et al., 2010) is wake-promoting. Deficient Hcrt signaling underlies narcolepsy (Chemelli et al., 1999; Lin et al., 1999; Thannickal et al., 2000), a sleep disorder characterized by fragmented sleep, degraded sleep–wake rhythms, and profound dysregulation of REM sleep. Hcrt signaling thus plays a critical role in the organization and consolidation of sleep–wake states.

Hcrt neurons project to several wake-promoting brain populations, including the locus coeruleus (LC; Peyron et al., 1998; Chemelli et al., 1999; Horvath et al., 1999). LC activation desynchronizes cortical activity and precedes transitions to waking, exhibiting a strongly wake-active, REM-silent firing profile (Aston-Jones and Bloom, 1981; Berridge and Foote, 1991; Takahashi et al., 2010). Optogenetic inhibition or activation of LC norepinephrine (NE) neurons increases or decreases the likelihood of sleep, respectively (Carter et al., 2010). Disruption of NE signaling via cell-type-specific LC lesions or knockout (KO) is reported to increase NREM sleep (González et al., 1998; Blanco-Centurion et al., 2004; Ouyang et al., 2004) or block wakefulness following arousing stimuli (Hunsley and Palmiter, 2004; Gompf et al., 2010), consistent with a role in maintenance of wakefulness. The LC expresses Hcrt receptor 1 (HcrtR1; Marcus et al., 2001) and Hcrt-1/orexin-A infusion into the LC increases LC neuron firing and promotes wakefulness (Hagan et al., 1999; Bourgin et al., 2000) in a HcrtR1-dependent manner (Soffin et al., 2002; Choudhary et al., 2014). Conversely, optogenetic LC inactivation blocks transitions to wakefulness following Hcrt neuron activation (Carter et al., 2012), indicating that the LC is important for Hcrt-induced wakefulness.

Hcrt neurons also strongly innervate histaminergic (HA) cells in the tuberomammillary nuclei (TMN) of the posterior hypothalamus (Peyron et al., 1998; Chemelli et al., 1999). TMN HA neurons express HcrtR2 (Marcus et al., 2001) and are excited by Hcrt peptides (Eriksson et al., 2001). HA is wake-promoting (Chu et al., 2004; Ramesh et al., 2004) and TMN HA neurons, like LC NE neurons, exhibit a wake-active, REM-off firing pattern (Takahashi et al., 2006). TMN HA lesions have relatively mild effects on sleep–wake states (Gerashchenko et al., 2004). However, mice unable to synthesize HA exhibit decreased wakefulness at lights-off, increased REM sleep time during the light phase, and short sleep latency in a novel environment (Parmentier et al., 2002; Anaclet et al., 2009). Wake promotion by Hcrt-1/orexin A is mediated in part through histaminergic neurotransmission (Huang et al., 2001). Thus, Hcrt inputs to the LC NE system and the TMN HA system are important pathways for Hcrt-induced wakefulness.

The dual Hcrt/orexin receptor antagonist (DORA) almorexant (ALM) blocks the excitatory effects of the Hcrt peptides at HcrtR1 and HcrtR2, decreasing wakefulness and increasing NREM and REM sleep time (Brisbare-Roch et al., 2007; Morairty et al., 2012). In contrast, zolpidem (ZOL; trade name Ambien) induces somnolence by activating GABA_A_ receptors, thereby causing widespread neuronal inhibition (Dang et al., 2011). ALM, but not ZOL, requires an intact basal forebrain (BF) for maximal hypnotic efficacy and induces neurochemical events associated with the transition to normal sleep (Vazquez-DeRose et al., 2014). These findings support the hypothesis that ALM induces sleep by selectively disfacilitating subcortical wake-promoting populations whereas ZOL acts via generalized inhibition throughout the brain. Here, we tested this hypothesis by selectively ablating the LC NE neurons or the TMN HA neurons using cell-type-specific saporin conjugates, and subsequently evaluating the efficacy of ALM and ZOL in lesioned and intact rats. We find that eliminating either HcrtR1 signaling in the LC or HcrtR2 signaling in the TMN yields similar effects on ALM-induced REM sleep without affecting NREM sleep time. Because a previous study (Vazquez-DeRose et al., 2014) found the converse effects after ablation of basal forebrain cholinergic neurons, these results support the concept that Hcrt neurotransmission influences distinct aspects of NREM and REM sleep at different locations in the sleep—wake regulatory network.

## Materials and Methods

### Animals

Male Sprague-Dawley rats (*n* = 25; 200–250 g; Harlan Laboratories) were housed in light-tight, sound-attenuated environmental chambers under constant temperature (22 ± 2°C, 50 ± 25% relative humidity) on a 12 h dark/light cycle with food and water *ad libitum*. All dosing procedures were performed under dim red light (<2 lux). All studies were conducted in accordance with the *Guide for the Care and Use of Laboratory Animals* and were approved by the Institutional Animal Care and Use Committee at SRI International.

### Chemicals

ALM was synthesized by the Medicinal Chemistry Laboratory at SRI International according to previously published methods (Koberstein et al., 2003, 2005). ZOL was purchased from IS Chemical. All drugs that were delivered orally were suspended and sonicated for 1 h in 1.25% hydroxypropyl methyl cellulose with 0.1% dioctyl sodium sulfosuccinate and 0.25% methylcellulose in sterile water [hereafter referred to as vehicle (VEH)]. All drug solutions were made on the day of the experiment and serially diluted to their final concentrations.

### Saporin lesions

Under isoflurane anesthesia, rats were placed into a stereotaxic apparatus (Kopf Instruments) and the skull was exposed. For LC lesions, rats were injected intracerebroventricularly with 10 μl of anti-dopamine beta hydroxylase-conjugated saporin (*n* = 8; DBH-SAP; 0.3 μg/μl; Advanced Targeting Systems; Wrenn et al., 1996; Wiley and Kline, 2000; Brightwell and Taylor, 2009) or sterile saline (*n* = 7; hereafter referred to as “Sham” rats) via a 26 gauge stainless steel injection cannula connected to a 10 μl Nanofil Hamilton syringe and a digitally controlled microinjector (World Precision Instruments) at −0.8 mm AP and +1.5 mm ML relative to bregma, and 3.3 mm below dura. The infusion volume and concentration were selected based on previously published methods and were verified in pilot studies. Injections lasted ∼10 min; the cannula was left in place for 5 min after the injection. For TMN lesions, rats were injected bilaterally with 250–350 nl of Hcrt2-saporin (*n* = 13; Hcrt2-SAP; 0.228 μg/μl; Advanced Targeting Systems; Gerashchenko et al., 2001, 2004) or sterile saline (*n* = 7) via glass micropipettes (inner tip diameter ∼30–50 μm) using a Picospritzer (Parker Hannifin) at −4.2 or −4.35 mm AP and ±0.8 mm ML relative to bregma, and 9.3 mm below dura. Injectate volume was measured via precalibrated marks on the barrel of the pipette. Injections lasted 5 min/side; the pipette was left in place for 5 min after the injection. Following SAP injections, rats were instrumented for EEG/EMG telemetry.

### Telemetry surgery

All rats were surgically implanted with a sterile abdominal transmitter (F40-EET, DSI) for continuous telemetric recordings of electroencephalograph (EEG), electromyograph (EMG), core body temperature (T_b_), and locomotor activity as described previously (Morairty et al., 2008, 2012). Briefly, the wires from the transmitter were subcutaneously channeled rostrally to the head. Two biopotential leads (EEG electrodes) were inserted into drilled holes over the dura (lead 1: +2.0 mm AP, +1.5 mm LM; lead 2: −7.0 mm AP, −2.0 mm LM; all coordinates relative to bregma) and affixed with dental acrylic. Two additional biopotential leads (EMG electrodes) were sutured into the neck musculature and closed with non-absorbable suture. Both DBH-SAP (Wrenn et al., 1996; Blanco-Centurion et al., 2004; Gompf et al., 2010) and Hcrt2-SAP (Gerashchenko et al., 2004) induce maximal degeneration by 12–14 d postinjection. Accordingly, animals were singly housed after surgery and allowed to recover undisturbed in their home cage for 3 weeks to allow sufficient time for SAP-induced neurodegeneration and sleep–wake behavior to stabilize prior to any recordings.

### Assessment of hypnotic efficacy in saporin-lesioned rats

Rats were kept in their home cages for the duration of the study in ventilated, light-tight, and sound-attenuated chambers in 12 h light/dark cycles. Prior to initiation of sleep recordings, animals were acclimated to handling for ∼1 week, and were dosed with VEH once per day for the last 3 d of the acclimation period. Animals were then left undisturbed for 2 d after acclimation was complete. Rats were administered ALM (30, 100, and 300 mg/kg), ZOL (10, 30, and 100 mg/kg), or VEH orally starting at lights-out (ZT 12) with at least 3 d between treatments to allow sufficient time for washout between doses. Drug treatments were balanced across lesion condition and treatment day, such that every dose was administered on each treatment day, and an approximately equal number of lesioned and sham rats received each dose on each treatment day. EEG was recorded for 24 h following dosing; the first 6 h following dosing was scored and analyzed (from ZT12 to ZT18).

To confirm the extent of lesions, rats were deeply anesthetized and transcardially perfused with heparinized 0.1 m phosphate-buffered saline followed by 4% paraformaldehyde. Brains were removed, postfixed in 4% paraformaldehyde, and then transferred to 30% sucrose until sectioning. Brains were sectioned at 40 µm on a freezing microtome. Free-floating sections containing the LC (bregma −9.16 mm to −10.30 mm) were incubated with 1% H_2_O_2_ for 15 min to quench endogenous peroxidase activity, followed by: (1) 1 h in blocking buffer containing 3% normal donkey serum, (2) overnight in mouse anti-DBH (1:100,000, MAB308, EMD Millipore), (3) 2 h in biotinylated donkey anti-mouse IgG (1:500; Jackson ImmunoResearch), and (4) 2 h in avidin–biotin complex (Vector Laboratories). DBH was visualized by reacting sections in 0.05% diaminobenzidine tetrahydrochloride and 0.01% H_2_O_2_ to form a brown reaction product. Sections were then mounted, dehydrated and coverslipped. To visualize HA neurons, sections containing the TMN (bregma −3.80 to −4.80 mm) were processed using a similar protocol that was modified as follows: (1) sections were incubated overnight in rabbit anti-adenosine deaminase (ADA; 1:20,000, ab176, EMD Millipore), followed by (2) 2 h in biotinylated donkey anti-rabbit IgG (1:500; Jackson ImmunoResearch).

**Figure 1.**
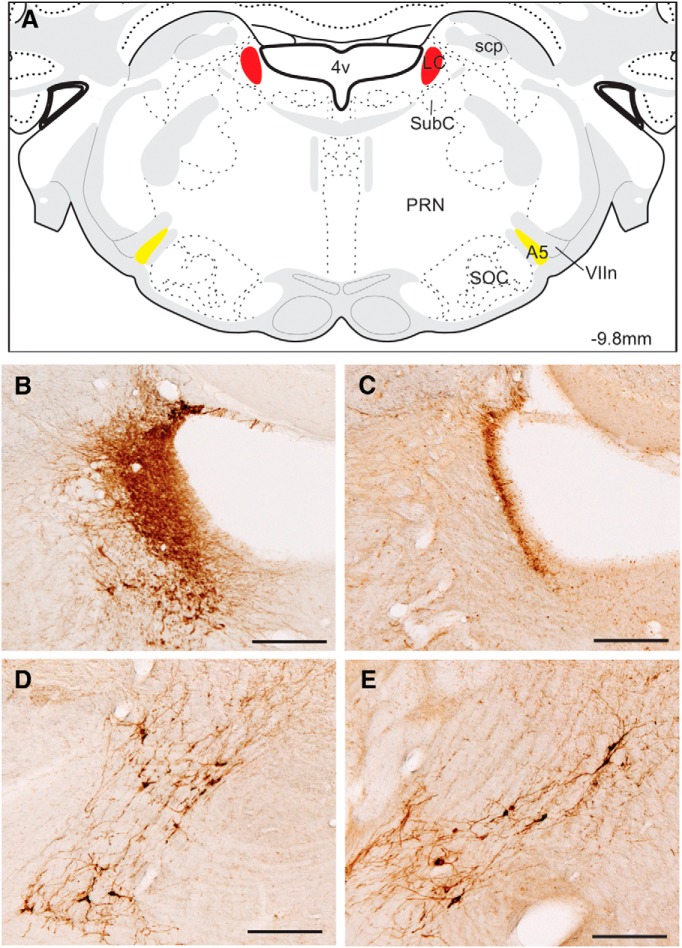
Characterization of DBH-SAP lesions. ***A***, Schematic showing location of LC NE neurons targeted by DBH-SAP infusions (red) and the more ventrally located A5 noradrenergic neurons (yellow). ***B***, DBH immunostaining of the LC in a Sham-injected rat shows densely-packed NE neurons, which were destroyed following DBH-SAP injections (***C***). By contrast, A5 neurons were intact in both Sham (***D***) and DBH-SAP-injected rats (***E***). 4v, Forth ventricle; A5, A5 NE group; PRN, pontine reticular nucleus; scp, superior cerebellar peduncle, SOC, superior olivary complex; SubC, subcoeruleus; VIIn, facial nerve. Scale bar, 200 μm. Adapted from Swanson (2004).

The extent of the LC was delineated by the fourth ventricle and other landmarks. The exact number of DBH-positive LC neurons could not be accurately counted in Sham rats because of the high density of these cells (Fig. 1*B*); accordingly, Sham rats were only scored for the presence of DBH-positive cells. In DBH-SAP injected rats, all residual DBH-positive cells in the LC region were counted. To evaluate the extent of TMN HA neuronal loss, ADA-positive neurons were counted in the dorsal (dTMN), ventral (vTMN), and caudal TMN (cTMN) subregions as identified previously (Ko et al., 2003; Parks et al., 2016). All neurons expressing ADA in each subregion were scored.

### EEG and EMG analyses and sleep–wake determinations

EEG and EMG were recorded via telemetry on a PC running Dataquest ART 3.1 (DSI). All recordings were manually scored off-line by a trained expert in 10 s epochs as Wake, NREM, or REM sleep using NeuroScore 2.1 (DSI). Any epochs that contained recording artifacts were tagged and excluded from spectral analyses. Individual state data were quantified as time spent in each state per 6 h. Latency to NREM and REM onset for each animal was calculated from the time of drug injection. Bouts were defined as a minimum of three consecutive epochs of wake or NREM, and two consecutive epochs of REM sleep.

### Statistical analyses

Latency to NREM and REM sleep, total time in each state (wake, NREM, REM), REM–NREM ratio, average bout duration, and total number of bouts for the 6 h following dosing were analyzed by a two-way ANOVA comparing lesion condition (between-subjects) and drug treatment (within-subjects). Bout architecture was further analyzed using a three-way mixed model ANOVA comparing the effects of lesion condition (between-subjects), drug treatment (within-subjects) and bout duration (within-subjects) on bout number. To assess fragmentation of arousal states, we included all sleep–wake bouts without a minimum bout length requirement. Significant main effects and interactions (*p* < 0.05) were subsequently analyzed with Bonferroni *post hoc* tests. In some cases, near-significant trends in the omnibus ANOVA were followed up with planned comparisons (*F* test), examining the effects of lesion at each drug dose; these planned comparisons are specified in the results. TMN lesion efficacy was assessed via a Student’s t-test. All statistical analyses were run using Statistica (Statsoft), except the *t* test and accompanying power analysis (Table 1, row n), which were run using R and G*Power, respectively.

**Table 1.** Statistical table

	**Data structure**	**Type of test**	**Observed power**
**a**	Normal distribution	2-factor mixed-model ANOVA	0.82
**b**	Normal distribution	2-factor mixed-model ANOVA	1.00
**c**	Normal distribution	2-factor mixed-model ANOVA	1.00
**d**	Normal distribution	2-factor mixed-model ANOVA	0.60 (lesion); 1.00 (drug)
**e**	Normal distribution	2-factor mixed-model ANOVA	0.98
**f**	Normal distribution	2-factor mixed-model ANOVA	0.99
**g**	Normal distribution	2-factor mixed-model ANOVA	0.61 (lesion); 1.00 (drug); 0.73 (interaction)
**h**	Normal distribution	2-factor mixed-model ANOVA	0.81
**i**	Normal distribution	2-factor mixed-model ANOVA	0.98
**j**	Normal distribution	2-factor mixed-model ANOVA	0.79 (lesion); 1.00 (drug)
**k**	Normal distribution	3-factor mixed-model ANOVA	1.00
**l**	Normal distribution	3-factor mixed-model ANOVA	1.00
**m**	Normal distribution	3-factor mixed-model ANOVA	0.98 (drug × lesion); 0.98 (bout × lesion)
**n**	Normal distribution	Student’s *t* test	1.00
**o**	Normal distribution	2-factor mixed-model ANOVA	1.00
**p**	Normal distribution	2-factor mixed-model ANOVA	0.99
**q**	Normal distribution	2-factor mixed-model ANOVA	1.00
**r**	Normal distribution	2-factor mixed-model ANOVA	1.00
**s**	Normal distribution	2-factor mixed-model ANOVA	0.79
**t**	Normal distribution	2-factor mixed-model ANOVA	1.00 (drug); 0.72 (interaction)
**u**	Normal distribution	2-factor mixed-model ANOVA	1.00
**v**	Normal distribution	2-factor mixed-model ANOVA	1.00
**w**	Normal distribution	3-factor mixed-model ANOVA	0.88
**x**	Normal distribution	2-factor mixed-model ANOVA	0.86

## Results

### LC lesion evaluation

Figure 1*A* shows the LC area targeted by the DBH-SAP lesions (red), as well as the approximate location of the nearby A5 NE neurons (yellow). In Sham-injected rats, the LC was clearly delineated by densely packed DBH-positive cells and fibers (Fig. 1*B*). The darkly stained neuropil and proximity of DBH-positive cells to each other made it difficult to accurately count individual cells. In DBH-SAP-injected rats, only a few scattered DBH-positive neurons were visible in the LC (Fig. 1*C*); bilateral counts in the LC revealed 15.75 ± 4.2 DBH-positive cells in SAP-treated rats, ranging from 2 to 38 neurons remaining in individual animals. In contrast, the more ventral A5 neurons were largely or entirely spared following DBH-SAP injections (Sham, Fig. 1*D*; DBH-SAP, Fig. 1*E*) as previously reported for a similar DBH-SAP dose (Wrenn et al., 1996; Brightwell and Taylor, 2009). All DBH-SAP-injected rats were thus considered to have complete LC lesions.

### LCx attenuates sleep induction by ALM

**Figure 2.**
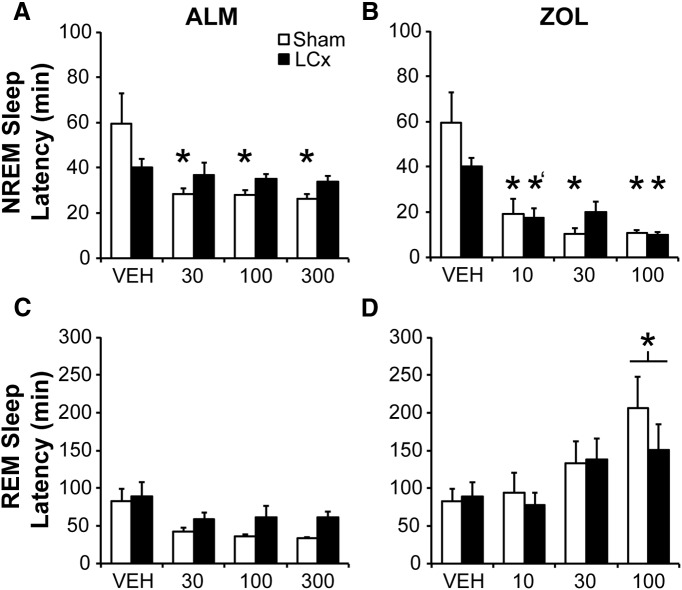
Latency to NREM (***A***, ***B***) and REM sleep (***C***, ***D***) following ALM (***A***, ***C***), and ZOL (***B***, ***D***) in LCx and Sham lesioned rats. Doses are mg/kg. *p < 0.05 versus vehicle; *'*p* < 0.06 versus vehicle.

Both ALM and ZOL (all doses) shortened the latency to NREM sleep compared with VEH in Sham rats, whereas only ZOL (10 and 100 mg/kg) was effective in LCx rats (interaction: *F*_(6,78)_ = 2.553, *p =* 0.026; Fig. 2*A*,*B*)_a_. Thus, LCx attenuated the ALM-induced but not the ZOL-induced decrease in NREM latency. Although LC lesions shortened NREM sleep latency following VEH from ∼60 to ∼40 min, the decrease was not significant compared to VEH-treated Sham rats likely because of the large variance in NREM latency among the Sham rats. Importantly, ZOL further decreased NREM latency from this baseline in LCx rats, whereas ALM only decreased NREM latency in Shams (Fig. 2*B*), indicating that lesioned rats could still respond to hypnotics. REM sleep latency was significantly increased by ZOL at 100 mg/kg independently of lesion status (main effect of drug: *F*_(6,78)_ = 12.58, *p* < 0.001; Fig. 2*C*,*D*)_b_.

### LCx attenuates REM sleep increases following ALM

**Figure 3.**
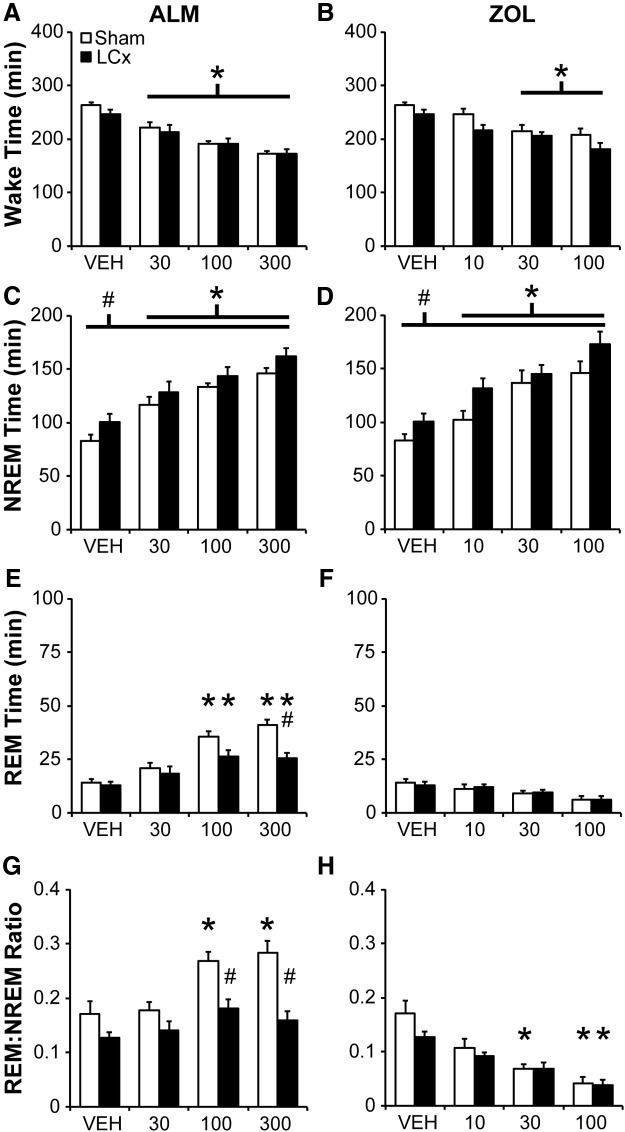
Total Wake (***A***, ***B***), NREM (***C***, ***D***) and REM (***E***, ***F***) sleep time, and the ratio of REM to NREM sleep (***G***, ***H***) following ALM (***A***, ***C***, ***E***, ***G***) and ZOL (***B***, ***D***, ***F***, ***H***) for 6 h following dosing in LCx and Sham lesioned rats. Doses are mg/kg. *p<0.05 versus vehicle; #*p*<0.05 (LCx vs Sham).

Both ALM (all doses) and ZOL (30, 100 mg/kg) decreased wake time for the 6 h period following dosing (ZT 12 to ZT 18) independent of lesion status (main effect of drug: *F*_(6,78)_ = 21.532, *p* < 0.001; Fig. 3*A*,*B*)_c_. NREM sleep time was increased by LC lesions (main effect of lesion: *F*_(1,13)_ = 5.722, *p* = 0.033)_d_ and by all doses of ALM and ZOL (main effect of drug: Fig. 3*C*,*D*; *F*_(6,78)_ = 18.821, *p* < 0.001)_d_, with no drug–lesion interaction (Fig. 3*C*,*D*). By contrast, ALM (100, 300 mg/kg) increased REM sleep time compared to VEH in both Sham and LCx rats, but this increase was attenuated in LCx compared with Sham rats at the ALM 300 mg/kg dose (interaction: *F*_(6,78_ = 4.439, *p* < 0.001; Fig. 3*E*,*F*)_e_. Similarly, ALM (100, 300 mg/kg) increased the ratio of REM to NREM sleep (REM–NREM) compared to VEH in Shams, but not LCx rats (interaction: *F*_(6,78)_ = 5.010, *p* < 0.001; Fig. 3*G*)_f_, such that REM–NREM was significantly attenuated in LCx rats compared to Shams following ALM (100 and 300 mg/kg). Although ZOL did not significantly affect REM sleep time (Fig. 3*F*), ZOL decreased REM–NREM in both Sham (30 and 100 mg/kg) and LCx rats (100 mg/kg; Fig. 3*H*). Thus, LCx blocked ALM-induced, but not ZOL-induced shortening of NREM sleep latency, attenuated the ALM-mediated increase of REM sleep time, and increased NREM sleep time independent of drug effects.

### LCx attenuates NREM and REM bout increases following ALM

**Figure 4.**
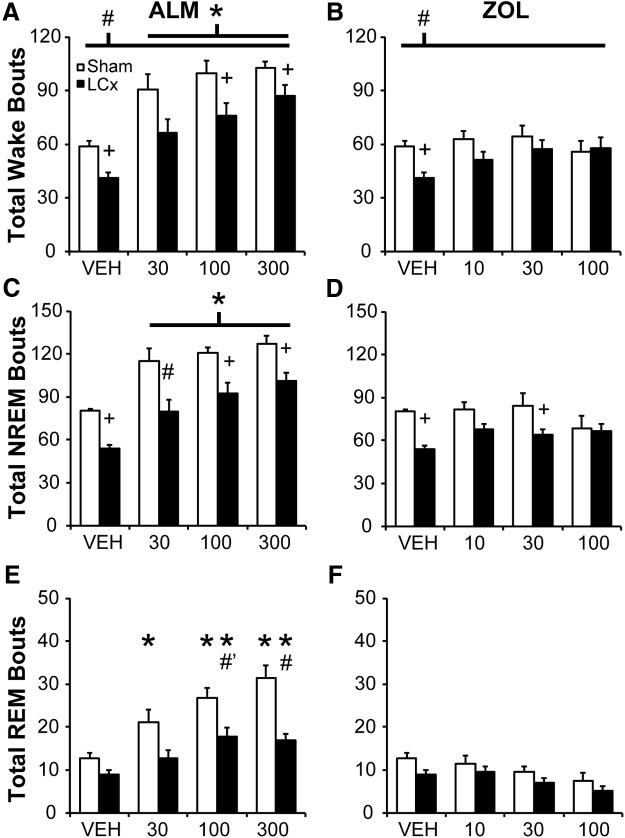
Total number of wake (***A***, ***B***), NREM (***C***, ***D***) and REM (***E***, ***F***) bouts for 6 h following ALM (***A***, ***C***, ***E***) and ZOL *(****B***, ***D***, ***F***) LCx and Sham lesioned rats. Doses are mg/kg. *p<0.05 versus vehicle; #*p*<0.05 (LCx vs Sham); #*p*<0.06 (LCx vs Sham); +*p*<0.05, paired comparison *F* test (LCx vs Sham).

LC lesions decreased the total number of wake bouts compared with Shams (main effect: *F*_(1,13)_ = 5.891, *p* = 0.030; Fig. 4*A*,*B*)_g_. ALM (all doses) increased the total number of wake bouts, whereas ZOL did not (main effect of drug: *F*_(6,78)_ = 29.152, *p* < 0.001; Fig. 4*A*,*B*)_g_. There was a borderline drug x lesion interaction (*F*_(6,78)_ = 2.136; *p* = 0.058)_g_; planned comparisons revealed that LCx rats had fewer wake bouts than Shams following VEH and ALM (100 and 300 mg/kg) but not after ZOL (Fig. 4*A*,*B*).

A similar effect was observed for the number of bouts of NREM sleep (interaction: *F*_(6,78)_ = 2.514, *p* = 0.028, Fig. 4*C*,*D*)_h_; ALM (all doses) significantly increased NREM bout number compared with VEH in all rats, but bout numbers were consistently lower in LCx compared with Sham rats at all doses (Fig. 4*C*). By contrast, ZOL tended to equalize NREM bout number between lesion conditions, especially at the highest dose (100 mg/kg; Fig. 4*D*).

ANOVA for the number of REM bouts revealed a significant drug x lesion interaction (*F*_(6,78)_ = 4.519; *p* < 0.001; Fig. 4*E*,*F*)_i_. ALM increased the number of REM bouts compared with VEH in both Sham (all doses) and LCx rats (100 and 300 mg/kg); however, LCx attenuated the ALM-induced increase at 300 mg/kg, with a borderline effect at the 100 mg/kg dose (Bonferroni, *p*=0.055). ZOL did not affect REM bout number in either Sham or LCx rats.

The mean duration of NREM bouts was independently increased by LC lesions (main effect of lesion: *F*_(1,13)_ = 8.848; *p* = 0.011)_j_ and by ZOL (100 mg/kg; main effect of drug: *F*_(6,78)_ = 6.734; *p* < 0.001)_j_, with no interaction between the factors (data not shown). There were no other effects on the mean duration of NREM, REM, or wake bouts.

### LCx preferentially attenuates short wake and NREM bouts following ALM

**Figure 5.**
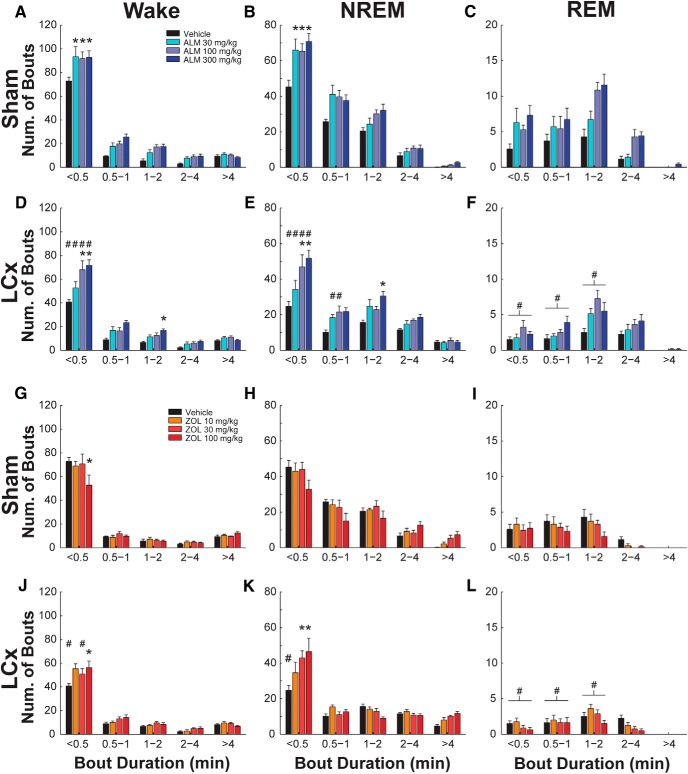
Number of wake (***A***, ***D***, ***G***, ***J***), NREM (***B***, ***E***, ***H***, ***K***), and REM (***C***, ***F***, ***I***, ***L***) bouts as a function of bout duration in Sham (***A***–***C***, ***G***–***I***) and LCx rats (***D***–***F***, ***J***–***L***) following ALM (***A***–***F***) or ZOL (***G***–***L***). *p<0.05 vs vehicle; #*p*<0.05 (LCx vs Sham).

We next asked whether changes in bout number were associated with changes in bout duration (Fig. 5). ALM preferentially increased the number of short (<0.5 min) wake bouts in both LCx and Sham rats (*F*_(24,312)_ = 3.814, *p* < 0.001; Fig. 5*A*,*D*)_k_, but LCx rats had fewer short wake bouts compared to Shams following VEH and ALM. Similarly, ALM also increased the number of short NREM bouts in both LCx and Sham rats (*F*_(24,312)_ = 4.739, *p* < 0.001; Fig. 5*B*,*E*)_l_, but LCx rats had fewer NREM bouts <1 min than Shams following VEH and ALM. In other words, ALM increased the number of short sleep–wake bouts, whereas LC lesions decreased the number of short bouts. By contrast, LC lesions had very little effect on wake and NREM bout architecture following ZOL. ZOL appeared to increase the number of long NREM bouts (>4 min) in both Sham (Fig. 5*H*) and LCx (Fig. 5*K*) rats. Although the *post hoc* comparisons were not statistically significant, the additional long bouts likely account for the significant increase in mean NREM bout duration under ZOL treatment.

There were significant drug × lesion (*F*_(6,78)_ = 4.473; *p* < 0.001)_m_ and bout x lesion interactions (*F*_(4,52)_ = 6.421; *p* < 0.001)_m_ that affected REM bout composition, but there was no three-way interaction between drug, bout, and lesion (Fig. 5*C*,*F*,*I*,*L*). As described above for total bout number, LCx attenuated the increase in REM bout number following ALM (100 and 300 mg/kg); Figure 5, *F* and *L*, show that the changes in REM bout number were distributed across short and long REM bouts and were observed following both ALM and ZOL.

### TMN lesion evaluation

**Figure 6.**
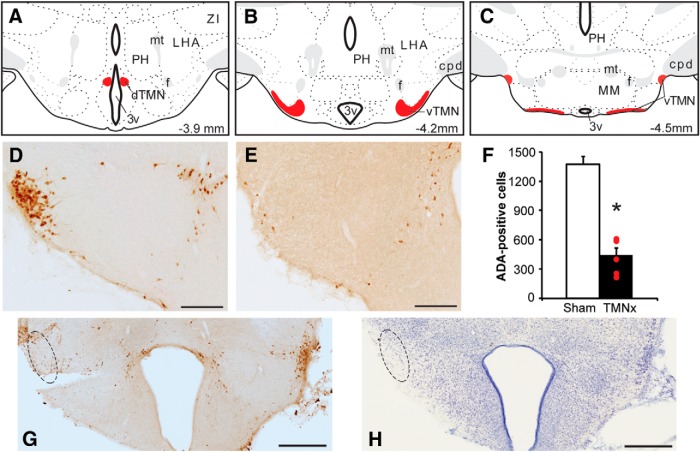
Characterization of Hcrt2-SAP lesions. ***A***–***C***, Schematic showing location of TMN HA neurons targeted by Hcrt2-SAP infusions. Panels adapted from Swanson (2004). ***D***, ***E***, HA-positive neurons in the dTMN and vTMN in a Sham-injected rat (***D***) and a Hcrt2-SAP injected rat (***E***). Photomicrographs depict the TMN at approximately the same rostrocaudal level as ***B***. ***F***, The TMNx group *(n* = 6) exhibited 445 ± 73 ADA-positive TMN cells, with individual lesions ranging from 216to 612 ADA-positive cells. ***G***, ***H***, Composite photomicrographs depicting ADA-immunostaining (***G***) and Nissl (***H***) in adjacent brain sections of a rat that was unilaterally injected with Hcrt2-SAP. Dotted lines in each panel indicate location of the vTMN in the injected hemisphere. 3v, Third ventricle; cpd, cerebral peduncle; f, fornix; LHA, lateral hypothalamic area; MM, medial mammillary nuclei; mt, mammillary tract; PH, posterior hypothalamic nucleus. Scale bars: ***D***, ***E***, 200 μm; ***G***, ***H***, 500 μm. *p < 0.05 (LCx vs Sham).

Figure 6*A*–*C* shows the posterior hypothalamic region targeted by the Hcrt2-SAP lesions, with the dorsal, ventral, and caudal TMN subgroups highlighted. ADA-immunostaining clearly visualized the HA neurons in Sham-injected rats (Fig. 6*D*). There were 1375 ± 78 ADA-positive cells in the TMN of Sham rats (combined count of dorsal, ventral, and caudal TMN). Hcrt2-SAP injections decreased ADA-immunostaining in the TMN (Fig. 6*E*,*G*). Inspection of Nissl-stained sections revealed that Hcrt2-SAP also destroyed some cells surrounding the TMN (Fig. 6*H*), as described in previous studies (Gerashchenko et al., 2004; Blanco-Centurion et al., 2007).

Of 13 Hcrt2-SAP injected rats, six exhibited substantial bilateral reductions in ADA-positive cell number (ranging from 16% to 45% of the Sham group mean). These six rats were used as the TMNx group. This TMNx group exhibited 445 ± 73 ADA-positive TMN cells, with individual lesions ranging from 216 to 612 ADA-positive cells, significantly fewer than in the Sham group (*t*_(11)_ = 8.731; *p* < 0.001_n_; Fig. *6F*, individual counts from each TMNx rat superimposed on the group mean). The remaining rats exhibited little to no ADA cell loss (>75% of Sham group mean), and were excluded from further analysis on the basis of being essentially unlesioned.

### TMNx attenuates REM sleep promotion by ALM

As in the LCx study described above, there was a significant main effect of drug treatment on NREM sleep latency (*F*_(6,66)_ = 11.243; *p* < 0.001)_o_ such that ZOL but not ALM significantly shortened the latency to NREM sleep (data not shown). There was also a significant main effect of drug treatment on REM sleep latency (*F*_(6,66)_ = 5.390; *p* < 0.001)_p_; whereas *post hoc* tests showed no significant changes compared with VEH, ALM tended to decrease REM sleep latency, whereas ZOL tended to increase it. Neither NREM nor REM sleep latency was affected by TMN lesion.

**Figure 7.**
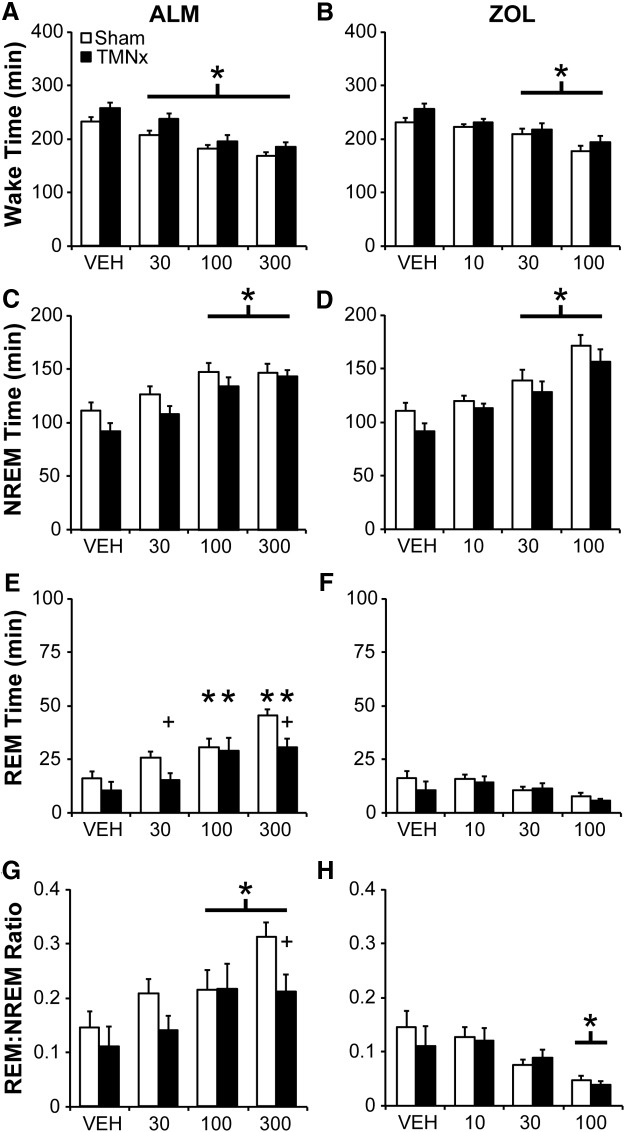
Total wake (***A***, ***B***), NREM (***C***, ***D***), and REM (***E***, ***F***) sleep time, and the ratio of REM to NREM sleep (***G***, ***H***) for 6 h following ALM (***A***, ***C***, ***E***, ***G***) and ZOL (***B***, ***D***, ***F***, ***H***) in TMNx and Sham lesioned rats. Doses are mg/kg. *p < 0.05 vs vehicle; +*p* < 0.05, paired comparison *F* test (TMNx vs Sham).

Consistent with the LCx study, both ALM and ZOL decreased wake time (main effect of drug: *F*_(6,66)_ = 29.346, *p* < 0.001; Fig. 7*A*,*B*)_q_ and increased NREM sleep time (main effect of drug: *F*_(6,66)_ = 27.612, *p* < 0.001; Fig. 7*C*,*D*)_r_, but with no main or interaction effect of TMN lesion. By contrast, ALM (100 and 300 mg/kg) increased REM sleep time compared with VEH in both Sham and TMNx rats (drug × lesion interaction: *F*_(6,66)_ = 2.436, *p* = 0.035; Fig. 7*E*,*F*)_s_. Pairwise comparisons of TMNx and Sham rats in each drug treatment condition revealed that TMNx rats had less total REM sleep time following ALM (30 and 300 mg/kg) compared to Shams, whereas there were no differences in REM sleep time between Sham and TMNx rats following ZOL (Fig. 7*E*,*F*). REM–NREM was significantly increased by ALM (100 and 300 mg/kg) and decreased by ZOL (100 mg/kg; main effect of drug: *F*_(6,66)_ = 28.419, *p* < 0.001; Fig. 7*G*,*H*)_t_. However, these drug effects were qualified by a borderline interaction effect (*F*_(6,66)_ = 2.131; *p* = 0.061)_t_, such that TMNx decreased REM–NREM compared to Shams following ALM (300 mg/kg; pairwise comparison, *p*< 0.05). Thus, TMNx affected ALM-induced REM sleep increases in a similar manner to that seen following LCx.

### TMNx attenuates increases in REM bout number following ALM

**Figure 8.**
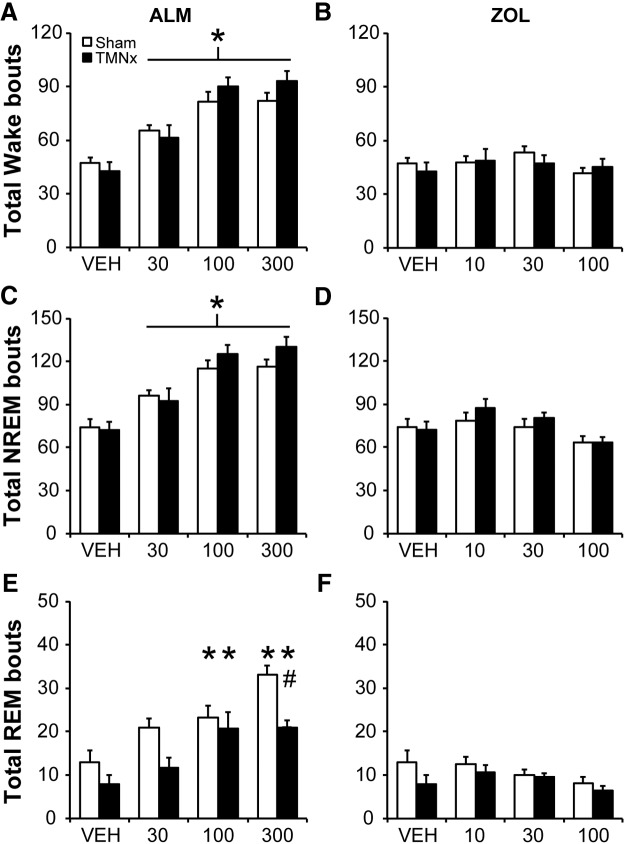
Total number of wake (***A***, ***B***), NREM (***C***, ***D***) and REM (***E***, ***F***) bouts for 6 h following ALM (***A***, ***C***, ***E***) and ZOL (***B***, ***D***, ***F***) in TMNx and Sham lesioned rats. Doses are mg/kg. *p < 0.05 versus vehicle; #*p* < 0.05 (TMNx vs Sham).

ALM (all doses) increased the total number of wake and NREM bouts compared to VEH (main effect of drug: wake, *F*_(6,66)_ = 48.670, *p* < 0.001_u_; NREM, *F*_(6,66)_ = 46.346, *p* < 0.001_v_) without an effect of TMN lesion (Fig. 8*A*,*C*). ZOL did not affect the total number of wake or NREM bouts (Fig. 8*B*,*D*). Further analysis of bout duration histograms showed that TMNx preferentially increased the number of short (<0.5 min) NREM bouts (bout × lesion interaction: *F*_(5,55)_ = 3.401; *p* = 0.010)_w_ with no additional influence of drug treatment (data not shown).

Whereas ALM (100 and 300 mg/kg) increased REM bout number in TMNx and Sham rats, TMNx significantly attenuated this increase at the highest dose of ALM (interaction: *F*_(6,66)_ = 2.860, *p* = 0.015; Fig. 8*E*)_x_. ZOL did not affect the total number of REM bouts in either lesion group (Fig. 8*F*). There were no additional effects of lesion or drug treatment on total REM, wake bout numbers, or on the distribution of bout numbers as a function of their duration (data not shown). Thus, TMNx attenuated the promotion of REM sleep by ALM primarily by decreasing the number of REM episodes, while increasing the number of short NREM sleep bouts independently of drug treatment.

## Discussion

The Hcrt system promotes wakefulness in part through excitation of wake-active monoaminergic populations, including the noradrenergic LC and histaminergic TMN. In this study, neurotoxic lesions of the LC NE neurons or TMN HA neurons selectively attenuated ALM-mediated increases in REM sleep but not NREM sleep. Furthermore, neither lesion altered efficacy of the GABA_A_ receptor agonist ZOL. These findings support the hypothesis that ALM promotes sleep via selective disfacilitation of subcortical arousal systems. In addition, these results highlight the important role of Hcrt input to monoaminergic populations in regulating REM sleep.

### Lesion efficacy

DBH-SAP infusion caused a near-complete loss of LC DBH-immunoreactive cells, with no signs of collateral or nonspecific damage. DBH-SAP is highly selective for LC NE neurons when delivered intracerebroventricularly or intraparenchymally (Wrenn et al., 1996; Blanco-Centurion et al., 2004). Medullary and pontine NE populations receive Hcrt projections (Baldo et al., 2003) and are suggested to play a role in the inhibition of REM sleep (Fenik et al., 2002; Rukhadze et al., 2008; Léger et al., 2009). Although we cannot rule out the possibility of collateral damage to these non-LC NE groups, the nearby A5 noradrenergic neurons appeared intact in our LCx rats following 3µg DBH-SAP, consistent with previous work showing that higher doses are required to lesion these populations (Wrenn et al., 1996). Furthermore, LC lesions increased NREM sleep but not REM sleep following VEH. We therefore conclude that the observed effects on ALM and ZOL efficacy are attributable to NE cell loss in the LC, and not to damage of neighboring noradrenergic or other cell groups.

In contrast to our LC lesions, our TMN-HA lesions were less extensive (55–84% ADA-positive cell loss in TMNx rats). However, more complete TMN lesions still leave basal sleep–wake parameters largely intact (Gerashchenko et al., 2004; Blanco-Centurion et al., 2007). Furthermore, significant deficits in motivated behavior were reported at a threshold of 45% ADA-positive cell loss (Valdés et al., 2010), whereas lesions comparable to ours (∼70% ADA-positive cell loss) impaired hippocampal LTP during walking (Luo and Leung, 2010) and enhanced isoflurane anesthesia induction (Luo and Leung, 2011), suggesting that our lesions, although not complete ablations, were sufficient to assess TMN function. On the other hand, we cannot rule out the possibility that loss of non-HA neurons expressing Hcrt receptors may contribute to our observed results. Inspection of Nissl-stained sections revealed that Hcrt2-SAP lesions destroyed (presumably non-HA) cells surrounding the TMN, as described in previous studies (Gerashchenko et al., 2004; Blanco-Centurion et al., 2007). Both wake-active and wake/REM-active neurons have been observed in this region (Steininger et al., 1999; Takahashi et al., 2006). The posterior hypothalamus also contains GABAergic neurons active in REM recovery sleep (Steininger et al., 1999; Sapin et al., 2010); destruction of such neurons could contribute to attenuated REM sleep in ALM-treated TMNx rats. However, such REM-active neurons appear to be relatively sparse in the immediate vicinity of the TMN (Sapin et al., 2010). These populations present great potential for future inquiry.

### LC and TMN influences on basal sleep and waking

LCx, but not TMNx, increased NREM sleep time overall, independently of drug treatment. Although some studies found no effect of LC NE ablation on basal NREM sleep time (Hunsley and Palmiter, 2003; Gompf et al., 2010), others observed increased NREM sleep in the dark phase or around lights-off (González et al., 1998; Blanco-Centurion et al., 2004; Ouyang et al., 2004), similar to our results obtained between ZT12 and ZT18, and consistent with the idea that the loss of LC NE signaling impairs the ability to maintain wakefulness. However, LC lesions did not impair the capacity of either drug to increase NREM sleep, as both ALM and ZOL increased NREM sleep time compared to VEH in both lesioned and unlesioned rats.

Neither LC nor TMN ablation affected basal REM sleep in the dark phase, as previously reported (González et al., 1998; Blanco-Centurion et al., 2004; Gerashchenko et al., 2004; Gompf et al., 2010), suggesting that multiple REM-suppressing or wake-promoting systems exist which compensate when one is lost (Blanco-Centurion et al., 2007). It is unlikely that such compensatory responses masked the response to ALM, as the response to another sleep-promoting agent (ZOL) was similar in LCx, TMNx, and Sham rats in both studies (eg, increased NREM sleep coupled with decreased REM sleep). Furthermore, neither lesion impacted NREM sleep increases following ALM, and LC lesions actually increased NREM sleep time (see previous paragraph). These targeted LC and TMN lesions therefore attenuated specific aspects of ALM-mediated sleep while leaving responses to ZOL intact, supporting the hypothesis that Hcrt antagonism acts at subcortical sleep–wake regulatory targets to promote sleep.

### The LC is a critical site of action for sleep induction by ALM

LC lesions blocked reductions in NREM sleep latency following ALM, whereas ZOL reduced NREM latency to a comparable extent in both lesioned and Sham rats, demonstrating that sleep latency changes following hypnotics are readily observed in LCx rats. LC lesions are thus unlikely to have obscured the sleep latency response to ALM. Although the persistence of rapid sleep onset following ZOL may be related to pharmacokinetics as well as site of action, the LC is clearly important for induction of NREM sleep by ALM. Increases in spontaneous LC firing rate anticipate transitions to wakefulness (Aston-Jones and Bloom, 1981; Takahashi et al., 2010); LC stimulation activates the cortex and hippocampus (Berridge and Foote, 1991) and promotes transitions from sleep to wakefulness (Carter et al., 2010). Ablation of NE neurons causes deficits in arousal following stress or novelty (Hunsley and Palmiter, 2004; Ouyang et al., 2004; Gompf et al., 2010). LC NE signaling is thus a key component of arousal, particularly in association with attention; these observations suggest that the LC is a critical site of action for ALM-mediated sleep induction.

LCx increased NREM sleep time and NREM bout duration while decreasing the number of short wake and NREM bouts, suggesting increased NREM consolidation. ALM-induced sleep is typically fragmented compared with ZOL-induced sleep in normal, healthy rodents (Morairty et al., 2012; Black et al., 2013). Although ALM increased the number of wake and NREM bouts in LCx as well as Sham rats, increased NREM bout numbers in LCx rats were still evident at the highest dose of ALM. By contrast, ZOL increased NREM bout duration while equalizing NREM bout numbers between LCx and Sham rats (Figs. 4*B*,*D*, 5*A*,*B*,*D,E*). LC ablation thus attenuated ALM- but not ZOL-induced changes in sleep architecture. Stimulating Hcrt neurons (Adamantidis et al., 2007; Choudhary et al., 2014) or infusing Hcrt-1 centrally (Piper et al., 2000; Morairty et al., 2011) or directly into the LC (Bourgin et al., 2000) promotes waking, whereas local application of HcrtR1 antagonists or optogenetic LC inhibition blocks transitions to wakefulness (Carter et al., 2012), suggesting that Hcrt-mediated wakefulness is highly dependent on the LC NE system. Our results support this concept by demonstrating that LC ablation impacts both the sleep-induction profile of ALM and its effects on sleep bout architecture.

By contrast, TMNx increased the number of short NREM bouts but had no effects on initiation or duration of NREM sleep in any drug condition. Like the LC NE neurons, HA neurons exhibit a wake-active, REM-off firing pattern (Takahashi et al., 2006), elevated HA levels are correlated with wakefulness (Chu et al., 2004; Ramesh et al., 2004), and mice lacking HA exhibit deficits in wakefulness at lights-off and in a novel environment (Parmentier et al., 2002)—all of which suggest that HA is important for arousal. Hcrt directly and indirectly excites TMN HA neurons (Eriksson et al., 2001; 2004; Schöne et al., 2014) and Hcrt infusion into the TMN promotes waking and induces cortical HA release (Huang et al., 2001). Although it was surprising that TMN lesions did not affect NREM latency or NREM sleep time, Hcrt2-SAP TMN lesions exhibited few effects on basal sleep in a previous study (Gerashchenko et al., 2004).

### Lesioning either LC or TMN attenuates ALM-induced REM sleep

Blockade of Hcrt signaling with ALM increased REM sleep in Sham rats, as previously reported in intact animals (Brisbare-Roch et al., 2007); lesioning either the LC NE or TMN HA neurons selectively attenuated the promotion of REM sleep by ALM. LC lesions significantly decreased both REM–NREM and REM bout number at 100 and 300 mg/kg, whereas these measures were only affected at the 300 mg/kg dose in TMNx rats (Figs. 7*E*,*G*, 8*E*), suggesting a less robust influence of TMN HA neurons on REM sleep compared to the LC NE neurons. Alternatively, surviving HA neurons in lesioned rats may have been sufficient to maintain normal histaminergic regulation of REM sleep. However, the common influence of either lesion on ALM-induced REM sleep suggests a specialized role for Hcrt signaling to both of these nuclei in regulating REM sleep.

The LC NE neurons exhibit a wake-active, “REM-off” firing profile (Takahashi et al., 2010). The LC inhibits cholinergic brainstem “REM-on” neurons (Hobson et al., 1975; McCarley and Hobson, 1975). The LC NE neurons are a major target of the Hcrt neurons (Peyron et al., 1998) and express HcrtR1 almost exclusively (Marcus et al., 2001). Local Hcrt-1/orexin-A infusion activates the LC and suppresses REM sleep (Bourgin et al., 2000), whereas HcrtR1 knockout (Mieda et al., 2011), systemic HcrtR1 antagonism (Smith et al., 2003; Morairty et al., 2012), or intra-LC HcrtR1 blockade blocks Hcrt-mediated REM suppression (Bourgin et al., 2000; Choudhary et al., 2014), and siRNA downregulation of HcrtR1 in the LC increases spontaneous REM sleep in the dark phase (Chen et al., 2010). HcrtR1-mediated Hcrt signaling thus powerfully regulates the REM-suppressing role of the LC NE neurons, although this effect may be more clearly observed following LC-specific manipulations rather than systemic treatments.

TMN HA neurons, which express HcrtR2 (Marcus et al., 2001), also exhibit a wake-active, REM-off firing profile (Takahashi et al., 2006). Mice unable to synthesize HA exhibit increased REM sleep time (Parmentier et al., 2002; Anaclet et al., 2009). HA inhibits hypothalamic melanin-concentrating hormone neurons (Parks et al., 2014) that have been implicated in REM sleep (Verret et al., 2003; Clément et al., 2012; Jego et al., 2013), and administration of either ALM or a HcrtR2 antagonist decreases extracellular HA levels in the LH (Dugovic et al., 2009), consistent with a role for Hcrt-HA signaling in REM sleep regulation. Thus, the acute blockade of excitatory Hcrt input by ALM would “disfacilitate” REM-off activity in the LC and the TMN, resulting in the downstream disinhibition of REM-active populations such as brainstem cholinergic neurons targeted by the LC or the hypothalamic MCH neurons targeted by HA, respectively.

Lesioning either the LC, which expresses only HcrtR1, or the TMN, which expresses only HcrtR2 (Marcus et al., 2001), produced similar effects on ALM efficacy. Recent studies differ regarding whether blockade of Hcrt signaling at one or both Hcrt receptors is critical for promoting sleep (Smith et al., 2003; Dugovic et al., 2009; Mang et al., 2012; Morairty et al., 2012; Steiner et al., 2013). However, deletion of either Hcrt receptor modulates REM sleep response following Hcrt1 infusion (Mieda et al., 2011), while coadministration of HcrtR1 and R2 antagonists elicited synergistic effects distinct from those of either drug administered alone (Dugovic et al., 2009). The sleep-promoting effects of Hcrt receptor antagonism may thus depend on the resulting balance between HcrtR1 and HcrtR2 activity (Hoyer et al., 2013). In this light, localized manipulations such as lesions could also significantly alter drug efficacy by eliminating critical points in the Hcrt signaling pathway. Such manipulations would depend as much on the anatomical site of action as the receptor type(s) expressed there. For example, REM sleep is increased by siRNA knockdown of either HcrtR1 in the LC (Chen et al., 2010) or HcrtR2 in the lateral pontomesencephalic tegmentum (Chen et al., 2013), whereas lesioning the basal forebrain, which expresses both Hcrt receptors (Marcus et al., 2001), attenuates ALM-induced NREM, but not REM sleep (Vazquez-DeRose et al., 2014). In the present study, eliminating either HcrtR1 signaling in the LC or HcrtR2 signaling in the TMN yielded similar effects on ALM-induced REM sleep, independently of the Hcrt receptor type expressed in each region. Indeed, the effects of ablating the LC, which is thought to respond to Hcrt exclusively via HcrtR1, suggest strongly that HcrtR1-mediated LC NE activity represents a critical pathway for ALM-mediated sleep induction and REM promotion. Together, these findings suggest that the site of action, not just the specific receptor or receptors targeted, is a key determinant of how Hcrt receptor antagonism facilitates sleep.

## Conclusions

DORAs, including ALM, promote sleep by blocking Hcrt signaling. In this study, lesions of the wake-promoting LC NE or TMN HA populations compromised the REM sleep-promoting efficacy of ALM, whereas previous work has shown that ALM, but not ZOL, requires an intact BF for maximum NREM-promoting efficacy (Vazquez-DeRose et al., 2014). Together, these studies indicate that Hcrt neurotransmission influences distinct aspects of sleep at different locations in the sleep–wake regulatory network. Furthermore, our results, particularly our finding that LC lesions attenuate ALM efficacy, suggest that site of action is at least as important a consideration for Hcrt antagonist efficacy as the receptor targeted. By selectively disfacilitating these subcortical wake-promoting populations, ALM effectively promotes sleep by eliciting neurochemical events consistent with the transition to normal sleep (Vazquez-DeRose et al., 2014). These properties are likely to underlie the findings that ALM also promotes sleep without negatively impacting cognitive performance (Morairty et al., 2014) and without globally blocking the capability for arousal (Parks et al., 2016).

## Synthesis

The decision was a result of the Reviewing Editor Rae Silver and the peer reviewers coming together and discussing their recommendations until a consensus was reached. A fact-based synthesis statement explaining their decision and outlining what is needed to prepare a revision is listed below. The following reviewer(s) agreed to reveal their identity: Thomas Scammell

With regard to the minor comments, the following amplifications/clarifications are requested.

1) They authors acknowledge that saporin likely kills more than just the TMN neurons, and it would be helpful to examine Nissl-stained sections to estimate the size of the affected region so one can assess the likelihood of whether some of the behavioral change is caused by injury to non-histamine neurons.

2) On average, about 32% of the TMN neurons remained in the "TMN lesioned" group. Is this enough to really assess the TMN? How does this compare to prior lesion papers?

3) The photomicrographs convey the key message but could still be improved (lighting is uneven, parts are dark).
